# A methylation PCR method determines *FMR1* activation ratios and differentiates premutation allele mosaicism in carrier siblings

**DOI:** 10.1186/s13148-016-0280-8

**Published:** 2016-12-01

**Authors:** Andrew G. Hadd, Stela Filipovic-Sadic, Lili Zhou, Arianna Williams, Gary J. Latham, Elizabeth Berry-Kravis, Deborah A. Hall

**Affiliations:** 1 Research and Technology Development, Asuragen, Inc., Austin, TX 78744 USA; 2 Department of Neurological Sciences, Rush University Medical Center, Chicago, IL USA; 3 Departments of Pediatrics and Biochemistry, Rush University Medical Center, Chicago, IL USA

**Keywords:** Fragile X, Methylation PCR, *FMR1*, Premutation, Activation ratio, X-chromosome inactivation

## Abstract

**Background:**

Epigenetic modifications of the fragile X mental retardation 1 (*FMR1*) gene locus may impact the risk for reproductive and neurological disorders associated with expanded trinucleotide repeats and methylation status in the 5′ untranslated region. *FMR1* methylation is commonly assessed by Southern blot (SB) analysis, yet this method suffers a cumbersome workflow and relatively poor sizing resolution especially for premutation allele characteristic for fragile X-associated disorders. In this study, a methylation PCR (mPCR) assay was used to evaluate correlations among genotype, epitype, and phenotype in fragile X premutation (PM) carrier women in order to advance the understanding of the association between molecular determinants and the presence of fragile X-associated tremor and ataxia (FXTAS).

**Results:**

Activation ratios (ARs) in 39 PM women were determined by mPCR and compared with SB analysis. ARs were distributed across a range of values including five samples with <20% AR and six with >80% AR. The two methods were correlated (*R* ^2^ of 0.87 and *F* test of <0.001), indicating that mPCR can measure AR in agreement with established assays. However, mPCR was unique in identifying novel and distinct patterns of methylation mosaicism in premutation carrier women, including seven sibling pairs that were assessed using FXTAS clinical rating scales. Of note, four of six pairs with defined age of onset for neurological signs showed ARs consistent with skewed activation of the pathogenic expanded allele. One subject with severe FXTAS had a mosaic full mutation allele identified using mPCR but not detected by SB analysis.

**Conclusions:**

We utilized a repeatable and streamlined methodology to characterize *FMR1* inactivation in premutation carrier women. The method was concordant with SB analysis and provided higher resolution information on allele and methylation mosaicism. This approach can facilitate the characterization of epigenetic factors influencing fragile X phenotypes in larger cohort studies that can advance understanding and treatment of fragile X-associated disorders.

**Electronic supplementary material:**

The online version of this article (doi:10.1186/s13148-016-0280-8) contains supplementary material, which is available to authorized users.

## Introduction

The expansion of trinucleotide cytosine-guanine-guanine (CGG) repeats and methylation status of the fragile X mental retardation 1 (*FMR1*) gene are implicated in a number of developmental, neurodegenerative, and reproductive disorders. Expansions exceeding 200 CGG repeats are associated with hypermethylation of the promoter region, transcriptional silencing of the gene, and reduction or absence of expression of the *FMR1* protein (FMRP), which result in fragile X syndrome (FXS). FXS, which affects about 1 in 4000 males and 1 in 8000 females, is the most common inherited cause of intellectual disability and the most common single-gene defect linked to autism spectrum disorders [1, 2]. *FMR1* alleles with 55–200 repeats are categorized as premutations (PM) and have a prevalence of 1/151 to 1/209 women and 1/430 to 1/468 men [3, 4]. Clinical disorders associated with PM alleles include fragile X-associated tremor/ataxia syndrome (FXTAS) [5] and fragile X-associated primary ovarian insufficiency (FXPOI) [6]. While fewer than 45 CGG repeats is considered a “normal” allele, a conservative expansion of the repeat region to 45–54 CGG repeats is designated as intermediate or gray zone, although some groups define the gray zone as 41–54 repeats [7]. Intermediate alleles have been associated with the Parkinson disease and other disorders [8]. The risks for these disorders and the potential severity of relevant phenotypes are typically more pronounced in males because of a single X chromosome. However, in females, epigenetic influences such as X-chromosome inactivation (XCI), may impact the risks, age of onset, and severity of FXS, FXTAS, or FXPOI, as for other X-chromosome disorders [9].

PM carrier females may have a greater risk for presenting with fragile X-associated phenotypes if the normal allele is preferentially methylated (inactive). Reports in the literature describe a relationship between the *FMR1* PM and XCI in sibling pairs with discordant phenotypes [10, 11]. A report by Johnston-MacAnanny et al. found no evidence for a role of XCI in a case of monozygotic twin sisters with a *FMR1* PM but a different phenotype for FXPOI [10]. In contrast, Bodega et al. present evidence for a direct correlation between the activation status of the PM *FMR1* allele and FXPOI manifestations from an assessment of three sibling pairs with similar *FMR1* expansions, but discordance for the FXPOI phenotype [11]. Several studies have suggested an association of XCI with symptoms in sisters with neurological signs or FXTAS [12, 13], but these results represent individual families and it is not clear if the findings can be generalized.

Discrepancies in the role or contribution of XCI in PM carrier phenotypes may be explained in part by the small size of the cohorts, degree of mosaicism, and the technical variability and limitations of the methodologies used. These studies have typically used Southern blot (SB) analysis to query the methylation status of the *FMR1* gene or XCI analysis using the human androgen receptor (HUMARA) gene. SB analysis uses restriction digestion to interrogate the relative allele size and proportional methylation of both X chromosomes at the *FMR1* locus. This method can inform relative activation between normal and premutation alleles but is low throughput and labor intensive. Moreover, SB analysis has low sizing resolution and detection sensitivity for shorter alleles compared to methylation PCR (mPCR) [14]. Comparatively, XCI analysis using the HUMARA gene relies on methylation-sensitive restriction digestion (e.g., HpaII), PCR, and capillary electrophoresis to differentiate X chromosomes polymorphic for a variable CAG repeat region (9 to 36 CAG repeats). Separation of alleles heterozygous for the number of repeats and quantification of relative peak heights provides a reference for XCI. This approach offers higher throughput and reproducible analysis of XCI than SB. However, as Amos-Landgraf et al. reported, approximately 20% of women may have confounded interpretation in cases of homozygosity or poorly resolved CAG repeat alleles [15]. Moreover, activation ratios derived from a distinct gene region are only surrogates for the activation status of the *FMR1* gene. In this study, we sought to establish more accurate and higher throughput methods for obtaining methylation status of the *FMR1* locus to examine the relationship with fragile X-associated phenotypes.

mPCR quantifies allele-specific methylation at the *FMR1* locus [14, 16] with a simplified workflow relative to SB analysis. The method is based on methylation-specific digestion using HpaII and comparison to a control-digested aliquot of the same DNA using PCR and capillary electrophoresis for simultaneous determination of repeat length and methylation status. Prior work [14, 16] established concordance for methylation status in a range of genotypes and full-mutation alleles in cell lines and clinical samples. In this study, we extend the use of mPCR to the assessment of *FMR1* activation ratios (AR) in female PM carriers to evaluate potential correlation with and improvements over SB analysis. AR analysis using mPCR was also applied to a preliminary cohort of sibling pairs with concomitant FXTAS rating scale data. mPCR represents a fundamental improvement in the analysis of activation ratios at the *FMR1* locus compared to current methods and provides additional information in the study and characterization of fragile X PM disorders.

## Materials and methods

### Clinical genomic DNA samples

Genomic DNA samples from female PM carriers were analyzed in this study. Informed consent from all participants was obtained under an Institutional Review Board approved procedures at Rush University Medical Center (RUMC). The DNA was isolated from peripheral blood, stored at −20 °C, and shipped anonymized to Asuragen, Inc., for analysis. The cohort included samples from seven sibling pairs that had been assessed for FXTAS as part of a larger study to determine the neurological and endocrine phenotype of PM carrier women. Examinations from all women were scored using the FXTAS Rating Scale (FXTAS-RS), to evaluate the motor signs of FXTAS, and women were evaluated using diagnostic criteria for FXTAS [17].

### Methylation analysis

**Fig. 1:**
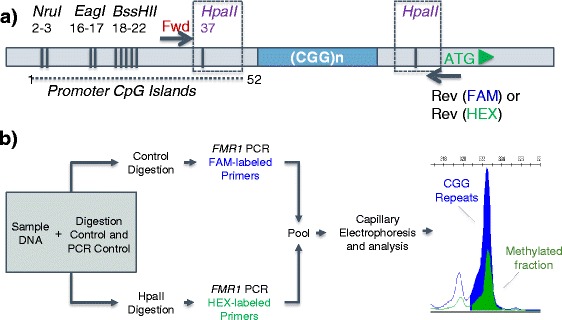
mPCR uses HpaII restriction digestion, two-color PCR and CE for determination of size and methylation. **a** Schematic diagram of restriction sites in the 5’ UTR of the *FMR1* gene. The HpaII sites flank the CGG repeat region. *Arrows* indicate location of the forward (mPCR Fwd) and reverse (mPCR Rev) PCR primers. **b** mPCR assay workflow. Percent methylation can be calculated from the normalized ratio of peak heights in the HEX and FAM channels

The AR of the normal *FMR1* allele was determined with AmplideX® *FMR1* mPCR reagents (Asuragen, Inc.) following manufacturer-recommended protocols and previously published methods [14, 16]. Briefly, genomic DNA was premixed with a reference control plasmid DNA, used to assess amplification efficiency, and digestion control plasmid DNA, used to determine digestion efficiency. This mixture was divided into two restriction enzyme reactions: (1) a methylation-sensitive restriction digestion and (2) a control digestion reaction. Products of the methylation-specific reaction were amplified using HEX-labeled PCR primers, and the products from the control digestion were amplified with FAM-labeled primers. PCR products from these separate reactions were pooled and analyzed using capillary electrophoresis (3500xL Genetic Analyzer, Thermo Fisher Scientific) for coincident determination of repeat length in the FAM channel and methylation status in the HEX channel. Repeat lengths were determined by correcting the size and mobility profile of CGG repeat DNA relative to the size ladder as previously described [16]. The percent methylation (%Me) for each peak_i_ was calculated as the ratio of peak heights normalized to the peak height of the reference control according to Eq. 1:

1 $$ \%{\mathrm{Me}}_i=\left(\frac{{\mathrm{Peak}}_{i,\mathrm{HEX}}}{{\mathrm{Ref}}_{\mathrm{HEX}}}\right)/\left(\frac{{\mathrm{Peak}}_{i,\mathrm{F}\mathrm{A}\mathrm{M}}}{{\mathrm{Ref}}_{\mathrm{FAM}}}\right)\times 100\% $$

where Peak_*i*, HEX_ is the signal height in the HEX channel corresponding the methylated fraction of Peak_*i*,FAM_ from the control digestion reaction and REF_HEX_ and REF_FAM_ to the peak heights of the PCR reference peak in the HEX and FAM channels, respectively. A schematic of the workflow and digestion sites of HpaII relative to EagI and NruI along with the representative two-color output of mPCR is shown in Fig. 1.

Repeatability of methylation determination was assessed using a pooled cell line control (Fragile X Process Control, Asuragen, Inc.) comprised of alleles representing a range of repeat lengths and methylation states. The repeat lengths (and expected methylation) of the alleles in this control were 18 (<10%), 30 (60 ± 10%), 32 (<10%), 56 (40 ± 10%), 85 (<10%), 116 (90 ± 10%), and >200 CGG (<10%). Results from 16 repeat measurements over three different days using two operators and instrument platforms were consistent with expected values (Additional file 1: Table S1).

### Calculation of the fragile X AR

The AR was determined using the percent methylation on the normal allele amplicon peak according to Eq. 2:

2 $$ {\mathrm{AR}}_{\mathrm{normal}\kern0.2em \mathrm{allele}}=1-\%{\mathrm{Me}}_{\mathrm{normal}\kern0.2em \mathrm{allele}} $$

Calculations for AR were made using the normal allele peak height to match results from SB analysis and to account for the broader distribution of peaks and methylation mosaicism observed in the PM allele.

### SB analysis

SB analysis of *FMR1* was performed at RUMC according to standard methods [18]. Genomic DNA samples were digested with *EcoR* I and *Eag* I (methylation-sensitive restriction enzymes), and blots were probed with [^32^P]-labeled StB12.3. Fragile X ARs for the PM allele were quantified by densitometric scanning of bands corresponding to unmethylated (active) DNA on the SB. ARs for the normal allele were calculated as the signal from the unmethylated portion of the normal band divided by total signal in the unmethylated portion of both the PM containing and normal bands.

## Results

### mPCR analysis provided similar and higher resolution information compared to SB analysis

**Fig. 2:**
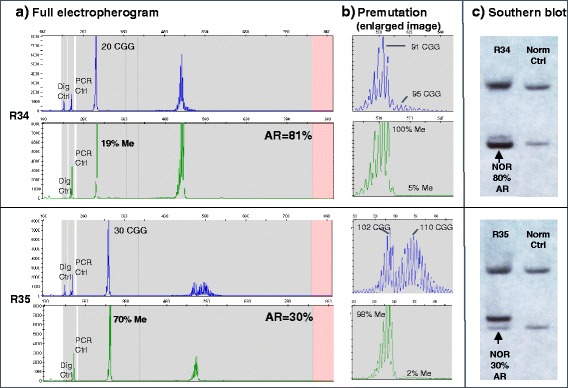
mPCR reveals ARs that correspond with SB results and distinct methylation patterns. **a** Full electropherogram profile for two representative samples (R34 and R35) showing amplicon peaks for the digestion control (Dig Ctrl), PCR control (PCR Ctrl) and normal and PM alleles. **b** An enlarged image of the PM region denotes distribution of sizes and methylation patterns. CGG repeat lengths were derived from the FAM channel (*blue* trace) and percent methylation from the HEX channel (*green* trace) with allele ratio shown for the normal peak. **c**) SB analysis of the same samples highlights similarity of allele ratio calculations for the normal allele with lower resolution compared to mPCR. The normal control is a female homozygous 30/30 CGG allele with partial methylation

We first examined the qualitative and quantitative differences between mPCR and SB analysis for premutation alleles. The distinctive features of an mPCR electropherogram for two samples plus their corresponding SB images are shown in Fig. 2. The full trace is shown in panel A, a highlight of the premutation allele amplicons is shown in panel B, and the SB images, with control bands, are shown in panel C. The electropherograms of signal versus size in base pairs for the allele amplicons from the control restriction digestion (FAM-labeled primers, blue) and from the methylation-sensitive restriction digestion (HEX-labeled primers, green) are shown. Residual signal in the HEX channel corresponds to the methylated fraction. These traces include the digestion control (expected to be <10% methylated) and the PCR reference control with signals in both FAM and HEX to be similar. The premutation allele amplicons were detected as a distribution of size and methylation mosaicism. Generally, and more prominently shown in sample R35 (Fig. 2), the premutation allele amplicons were fully methylated in the shorter or leading allele amplicons and unmethylated in the longer or trailing allele amplicons. In SB, partial methylation status was determined as the relative density of separately zoned bands detected within the unmethylated (<5.2 kb) region of the gel. These bands were unresolved in the methylated region (>5.2 kb) of the gel.

The two samples, highlighted in Fig. 2, provided a good example of different activation ratios and premutation allele resolution. The normal allele in sample R34 was preferentially unmethylated with an AR of 81% compared to sample R35, which had a more methylated normal allele and correspondingly lower activation ratio of 30%. These results were proportional to the relative density of the normal allele bands in the Southern blot image albeit with less resolution between the normal and premutation bands in the methylated zone of the gel. The majority of the premutation peak for R34 was expected to be methylated as indicated by the normal allele AR. While there was evidence of size and methylation mosaicism for the premutation allele, the majority of the allele amplicon peak area was detected in both the FAM and HEX channels corresponding to higher methylation for this allele (Fig. 2, panel B). For sample R35, the premutation peak profile was more distributed between methylated and unmethylated peaks. This sample had a lower AR with expected lower methylation in the premutation allele. This information was not evident using lower resolution SB analysis wherein subtle distributions of unmethylated and methylated components of the premutation allele are detected in separate regions of the gel. Because of the lower resolution in SB and the more distributed peak profile of the premutation allele using mPCR, activation ratios were calculated from the normal allele to make further comparisons between sample sets.

### Activation ratios determined using mPCR and SB were highly concordant

We next evaluated the ability of mPCR to determine ARs on a broader cohort representing distinctive ranges of low, partial, and high activation ratios. A summary of the sample cohort, CGG repeat lengths, and activation ratios between methods is provided in Additional file (Additional file 1: Table S2). Normal alleles ranged from 20 to 41 CGG repeats with an average of 29 CGG. Primary PM allele repeat lengths ranged from 58 to 120 CGG and included samples with mosaicism in the higher PM and low FM range. Normal allele ARs were distributed across a range of values including five samples with <20% AR and six with >80% AR.

**Fig. 3:**
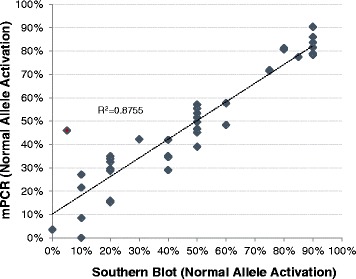
Normal allele ARs are highly concordant between mPCR and SB analysis. The ARs of 39 samples were assessed by mPCR and SB. The SB results are grouped in 10% increments compared to 1% increments using mPCR. ARs were detected across the range of mostly methylated (AR<20%> and mostly unmethylated (AR>80%)

ARs for the normal allele were compared between methods and plotted as percent activation (Fig. 3). Even with reporting differences of 1% increments for mPCR and 10% increments for SB analysis, the data were well correlated (*R* ^2^ of 0.87 and *F* test of <0.001) and supported a high level of agreement between both methods. Notably, unmethylated alleles with less than 20% methylation by SB analysis tended to report in higher ranges using mPCR. For example, two samples with 10% methylation by SB were 22 and 28% by mPCR. Another sample with 5% methylation by SB, confirmed on repeated blots, was detected at 46% by mPCR, a consistent result obtained on repeated measurements at both sites. The discordant result for this sample may be attributed to differences in restriction sites (as shown in Fig. 1) or to the lower resolution of SB for mostly unmethylated normal alleles (e.g., Fig. 2, panel C).

### mPCR reveals distinctive premutation allele patterns in sibling pairs

**Fig. 4:**
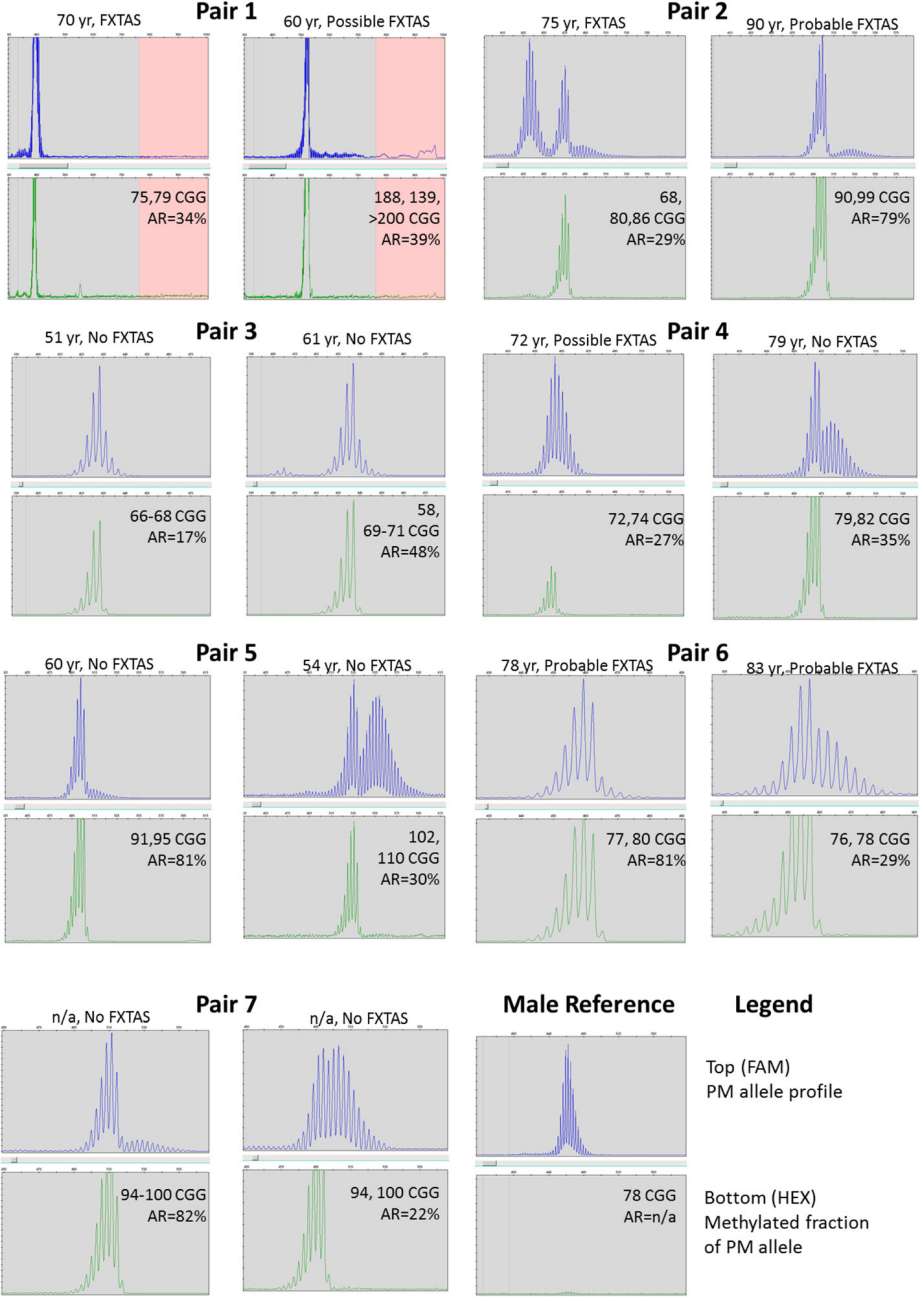
mPCR differentiates methylation mosaicism in PM alleles for sibling pairs. PM amplicon analysis from 7 sibling pairs showing CGG profile in the FAM (*top* panels, *blue* trace) and methylation fraction in the HEX (*bottom* panels, *green* trace) channels with allele ratio (AR), age of onset of neurological signs in years old (yo) and indication of FXTAS status. Methylation profiles showed predominantly methylated shorter amplicons and unmethylated fraction for longer amplicons. Within profile pairs, the relative ratio of methylated to unmethylated peaks were distinctive. An unmethylated male reference allele is shown to illustrate the distribution of allele peaks without a methylated fraction. Only premutation allele sizes as peak maxima are listed, ARs were determined from the percent methylation of the normal allele (not shown)

Due to the high concordance between mPCR and SB analysis, we considered if the additional information available using mPCR could be potentially linked to FXTAS phenotypes in sibling pairs. Results from the SB, mPCR, and FXTAS rating scale for the seven sibling pairs are listed in Table 1. Distributions of amplicon peak patterns and levels of methylation mosaicism could vary significantly between sisters (Fig. 4). In an extreme case, one sister, R27, had a minor mosaic full-mutation allele that was not detected by SB analysis (pair 1). In other cases, the proportion of active, unmethylated PM peaks was distinct between siblings (pairs 4 and 5). This level of methylation mosaicism may indicate relative activity of the PM allele or other features linked to phenotype. One sibling pair without neurological signs, pair 7, is included as a case control. A typical male premutation allele which lacks methylation, as expected, is included for reference.

**Table 1:** Sibling pairs and neurological phenotypes associated with repeat length and activation ratio

Pair	Neurological phenotype	Onset of neurological signs	CGG repeats	Activation ratio (SB)	Activation ratio (mPCR)	FXTAS motor rating scale	Neuro-imaging	FXTAS
1	Kinetic tremor, gait ataxia, parkinsonism	70	20/75, 79	20%	34%	20	Hyperintensities in white matter, midline pons	Definite FXTAS
	Mild gait ataxia	60	23/118, 139, >200	50%	39%	7	Hyperintensities (mild) in white matter	Possible FXTAS
2	Kinetic tremor, gait ataxia, parkinsonism	75	25/68, 80, 86	20%	29%	44	Hyperintensities in deep white matter, brainstem, cerebellum	Definite FXTAS
	Kinetic tremor, gait ataxia, parkinsonism	90	25/90, 99	90%	79%	74	N/A	Probable FXTAS
3	Numbness, dystonia of feet	51	27/66, 68	20%	16%	1	N/A	No
	Numbness of feet	61	27/58, 69, 71	50%	45%	4	N/A	No
4	Mild kinetic tremor, falls	72	30/72, 74	10%	27%	15	Hyperintensities (mild) in white matter	Possible FXTAS
	Mild kinetic tremor	79	30/79, 82	40%	35%	7	N/A	No
5	Mild kinetic tremor, mild gait ataxia	60	20/91, 95	80%	81%	12	N/A	Probable FXTAS
	Kinetic tremor, falls, dystonia	54	30/102, 110	20%	30%	7	N/A	No
6	Kinetic tremor, gait ataxia, parkinsonism	78	29/77, 80	80%	81%	14	N/A	Probable FXTAS
	Kinetic tremor, gait ataxia, parkinsonism	83	32/76, 78	40%	29%	13	N/A	Probable FXTAS
7	None	N/A	30, 94, 100	90%	82%	2	N/A	No
	None	N/A	30, 94, 100	10%	22%	4	N/A	No

Summary of phenotype, age of onset of neurological signs and results linking genotype, activation ratio by Southern blot (SB) and mPCR analysis and rating information for fragile X tremor and ataxia syndrome (FXTAS) by sibling pair

With regard to the association of these epigenetic patterns to FXTAS, the results were more varied, lacking direct association. In four of the seven sister pairs (pairs 2–5), the age of onset of neurological signs was earlier in the sister with the lower AR of the normal allele (Table 1). In the first of the three exceptions (pair 1), the CGG repeat size was higher in the sister with earlier age of onset and the ARs between the two were similar. In pair 6, age of onset was very close despite varying AR. Pair 7 is not yet affected, but the sisters were younger than 40. Many of the women did not have available neuroimaging, which was not required or collected in the study, and a more definitive diagnosis of FXTAS could not be made. Even though the sample size in this cohort was too small to determine whether there was an association between FXTAS and AR, the use of mPCR provided ARs, CGG repeat length, and additional layers of information consistent with the goals of understanding links between genotype and phenotype.

## Discussion

The ability to characterize epigenetic influences on *FMR1* gene expression can help improve understanding of risk, severity, and potential treatments for fragile X-associated disorders. In this study, we describe a simple yet powerful method for obtaining methylation status of the *FMR1* promoter region and compared results to SB analysis across a cohort of female PM carriers that included a FXTAS subgroup. Our findings demonstrate that mPCR is a reproducible, quantitative, and accurate method for the assessment of individual alleles and that the method can assess the activation status of the X chromosome. mPCR assay results strongly correlated with independent SB analyses and offered a number of advantages over this standard method, including a more streamlined workflow, higher resolution to detect similarly sized alleles, and a more refined quantification of the AR in PM alleles. The workflow for mPCR takes a total of ~8 h and 4 steps compared to an ~68 h turnaround time and multiple steps associated with SB analysis [19]. This approach could be used to support larger studies and assessment of novel epigenetic features that influence important phenotypes linked to the *FMR1* gene.

The use of mPCR revealed unusual methylation mosaic patterns in the expanded allele of PM carriers. Female PM alleles were generally characterized with a leading peak profile of predominantly methylated peaks and a trailing peak profile of predominantly unmethylated alleles [14]. The differential size and relative abundance of these peak patterns varied between carriers with equally sized PM alleles and between sibling pairs (Fig. 4). Males, with PM characterized by an unmethylated single group of peaks (Fig. 4), lack this mosaic pattern [14, 16]. A similar expanded and biphasic profile was also identified using a different technique in a mouse model of fragile X PM alleles [20]. In mice positive for a mutation in a methylation repair gene, *MSH2*, PM alleles had an expanded and biphasic profile. Mice null for this *MSH2* mutation had single distribution peaks. Results in these mouse models suggest somatic instability and contributions from other genetic risk factors involving methylation and transcription repair genes. mPCR can reveal these mosaic expansion and methylation patterns that might be indications of instability and/or risk factors for disease severity.

mPCR provides a complementary approach to recent advances in DNA sequencing technologies for the assessment of hydroxymethylation [21]. Direct sequencing provides an informative means to query the epigenetic status of the gene region without amplification or bias introduced through pre-treatment of the DNA with bisulphite. Methylation and hydroxymethylation could be directly inferred and mapped to specific CpG islands. In another study, a comprehensive survey of methylation and hydroxymethylation was used to characterize eight males with FXS compared to controls [22]. The authors report reciprocal effects of hydroxymethylation on gene expression compared to methylation and links to fragile X phenotype within a small cohort. In both of these techniques, application to female samples would have confounding information of epigenetic factors averaged across both the normal and expanded allele. The use of mPCR allows differentiation of allele size and status and identification of potential somatic instability within the expanded allele.

We applied mPCR within a small cohort of sibling pairs to determine if *FMR1* AR correlated with neurological signs and FXTAS. The majority of the sisters (4/7) had age of onset of neurological signs associated with AR. Of the three exceptions, one pair was likely too young to manifest FXTAS. Another had similar AR ratios, but different PM allele sizes. This may suggest that in the majority of sister pairs, a combination of CGG repeat size and AR could be a prognostic factor for the age of onset of FXTAS. However, CGG and AR were not informative for a sibling pair with milder FXTAS signs. Extending this work to a larger number of PM carrier women is warranted to determine if AR, CGG length, or both are associated with phenotype or prognosis. Additional epigenetic features, such as hydroxymethylation and factors that influence preferential mosaicism and activation of the PM allele, should also be assessed for their utility to increase predictive accuracy. The ability to associate epigenetic factors and activation ratios with clinical phenotypes is an important goal in clinical genetic counseling for women at risk of FXTAS.

## Additional file

Additional file 1:
**Table S1.** CGG repeat lengths and methylation analysis of a pooled positive control including 18, 30, 32, 56, 85, 116 and >200 CGG. **Table S2.** Cohort panel distribution of allele sizes and ARs determined using mPCR compared to the activation ratio from Southern blot analysis. (PDF 445 kb)

## Abbreviations

AR: Activation ratio; referring to the normal allele unless otherwise specified
Ctrl: Control (used in figure captions)
*FMR1*: Fragile X mental retardation-1
FXPOI: Fragile X-associated primary ovarian insufficiency
FXTAS: Fragile X-associated tremor-ataxia syndrome
mPCR: Methylation PCR (specifically, 2-color PCR with capillary electrophoresis)
*MSH2*: MutS homologue 2
SB: Southern blot
XCI: X-chromosome inactivation

## References

[1] Sherman S, Pletcher BA, Driscoll DA (2005). Fragile X syndrome: diagnostic and carrier testing. Genet Med.

[2] Wang LW, Berry-Kravis E, Hagerman RJ (2010). Fragile X: leading the way for targeted treatments in autism. Neurotherapeutics.

[3] Seltzer MM, Baker MW, Hong J, Maenner M, Greenberg J, Mandel D (2012). Prevalence of CGG expansions of the FMR1 gene in a US population-based sample. Am J Med Genet B Neuropsychiatr Genet.

[4] Tassone F, Iong KP, Tong TH, Lo J, Gane LW, Berry-Kravis E, Nguyen D, Mu LY, Laffin J, Bailey DB, Hagerman RJ (2012). FMR1 CGG allele size and prevalence ascertained through newborn screening in the United States. Genome Med.

[5] Jacquemont S, Hagerman RJ, Leehey MA, Hall DA, Levine RA, Brunberg JA, Zhang L, Jardini T, Gane LW, Harris SW (2004). Penetrance of the fragile X-associated tremor/ataxia syndrome in a premutation carrier population. JAMA.

[6] Allingham-Hawkins DJ, Babul-Hirji R, Chitayat D, Holden JJ, Yang KT, Lee C, Hudson R, Gorwill H, Nolin SL, Glicksman A (1999). Fragile X premutation is a significant risk factor for premature ovarian failure: the International Collaborative POF in fragile X study—preliminary data. Am J Med Genet.

[7] Loesch DZ, Bui QM, Huggins RM, Mitchell RJ, Hagerman RJ, Tassone F (2007). Transcript levels of the intermediate size or grey zone fragile X mental retardation 1 alleles are raised, and correlate with the number of CGG repeats. J Med Genet.

[8] Debrey SM, Leehey MA, Klepitskaya O, Filley CM, Shah RC, Kluger B, Berry-Kravis E, Spector E, Tassone F, Hall DA (2016). Clinical phenotype of adult fragile X gray zone allele carriers: a case series. Cerebellum..

[9] Plenge RM, Stevenson RA, Lubs HA, Schwartz CE, Willard HF (2002). Skewed X-chromosome inactivation is a common feature of X-linked mental retardation disorders. Am J Hum Genet.

[10] Johnston-MacAnanny EB, Koty P, Pettenati M, Brady M, Yalcinkaya TM, Schmidt DW. The first case described: monozygotic twin sisters with the fragile X premutation but with a different phenotype for premature ovarian failure. Fertil Steril. 2011;95:2431 e2413-2435 10.1016/j.fertnstert.2011.01.039 21300345

[11] Bodega B, Bione S, Dalpra L, Toniolo D, Ornaghi F, Vegetti W, Ginelli E, Marozzi A (2006). Influence of intermediate and uninterrupted FMR1 CGG expansions in premature ovarian failure manifestation. Hum Reprod.

[12] Zuhlke C, Budnik A, Gehlken U, Dalski A, Purmann S, Naumann M, Schmidt M, Burk K, Schwinger E (2004). FMR1 premutation as a rare cause of late onset ataxia—evidence for FXTAS in female carriers. J Neurol.

[13] Berry-Kravis E, Potanos K, Weinberg D, Zhou L, Goetz CG (2005). Fragile X-associated tremor/ataxia syndrome in sisters related to X-inactivation. Ann Neurol.

[14] Chen L, Hadd AG, Sah S, Houghton JF, Filipovic-Sadic S, Zhang W, Hagerman PJ, Tassone F, Latham GJ (2011). High-resolution methylation polymerase chain reaction for fragile X analysis: evidence for novel FMR1 methylation patterns undetected in Southern blot analyses. Genet Med.

[15] Amos-Landgraf JM, Cottle A, Plenge RM, Friez M, Schwartz CE, Longshore J, Willard HF (2006). X chromosome-inactivation patterns of 1,005 phenotypically unaffected females. Am J Hum Genet.

[16] Grasso M, Boon EM, Filipovic-Sadic S, van Bunderen PA, Gennaro E, Cao R, Latham GJ, Hadd AG, Coviello DA (2014). A novel methylation PCR that offers standardized determination of FMR1 methylation and CGG repeat length without southern blot analysis. J Mol Diagn.

[17] Leehey MA, Berry-Kravis E, Goetz CG, Zhang L, Hall DA, Li L, Rice CD, Lara R, Cogswell J, Reynolds A (2008). FMR1 CGG repeat length predicts motor dysfunction in premutation carriers. Neurology.

[18] Rousseau F, Heitz D, Biancalana V, Blumenfeld S, Kretz C, Boue J, Tommerup N, Van Der Hagen C, DeLozier-Blanchet C, Croquette MF (1991). Direct diagnosis by DNA analysis of the fragile X syndrome of mental retardation. N Engl J Med.

[19] Elias MH, Ankathil R, Salmi AR, Sudhikaran W, Limprasert P, Zilfalil BA (2011). A new method for FMR1 gene methylation screening by multiplex methylation-specific real-time polymerase chain reaction. Genet Test Mol Biomarkers.

[20] Zhao XN, Usdin K (2015). The transcription-coupled repair protein ERCC6/CSB also protects against repeat expansion in a mouse model of the fragile X premutation. Hum Mutat.

[21] Pham TT, Yin J, Eid JS, Adams E, Lam R, Turner SW, Loomis EW, Wang JY, Hagerman PJ, Hanes JW (2016). Single-locus enrichment without amplification for sequencing and direct detection of epigenetic modifications. Mol Genet Genomics.

[22] Brasa S, Mueller A, Jacquemont S, Hahne F, Rozenberg I, Peters T, He Y, McCormack C, Gasparini F, Chibout SD (2016). Reciprocal changes in DNA methylation and hydroxymethylation and a broad repressive epigenetic switch characterize FMR1 transcriptional silencing in fragile X syndrome. Clin Epigenetics.

