# Supplementary material for: A methylation PCR method determines FMR1 activation ratios and differentiates premutation allele mosaicism in carrier siblings
Source: Clin Epigenetics. 2016 Dec 1;8:130. doi: 10.1186/s13148-016-0280-8 (PMC5131543; doi:10.1186/s13148-016-0280-8)
Supplement: Additional file 1: — Table S1. CGG repeat lengths and methylation analysis of a pooled positive control including 18, 30, 32, 56, 85, 116 and >200 CGG. Table S2. Cohort panel distribution of allele sizes and ARs determined using mPCR compared to the activation ratio from Southern blot analysis. (PDF 445 kb) [file 13148_2016_280_MOESM1_ESM.pdf]

A methylation PCR method determines *FMR1* activation ratios and differentiates premutation allele mosaicism in carrier siblings

***Authors:** Andrew G. Hadd[1], Stela Filipovic-Sadic[1], Lili Zhou[2], Arianna Williams[2], Gary J. Latham[1], Elizabeth Berry-Kravis[2], and Deborah A. Hall[2].

Affiliations: [1]Asuragen, Inc., Austin, TX; [2]Rush University Medical Center, Chicago, IL

SUPPLEMENTAL DATA

Supplemental Table S1. Analysis of a positive control for CGG repeat and percent methylation. Distribution of observed CGG repeat lengths and percent methylation for allele amplicons across 2 operators and 2 instruments are listed. The target distribution range reflects acceptable ranges (as ± range) and observed methylation percentage is included.

		Target Allele Amplicons													
		18 ± 1 CGG		30 ± 1 CGG		32 ± 1 CGG		56 ± 1 CGG		85 ± 3 CGG		115 ± 3 CGG		>200 CGG	
Instrument	Operator	CGG	% Me	CGG	% Me	CGG	% Me	CGG	% Me	CGG	% Me	CGG	% Me	CGG	% Me
3130xl	1	18	2	30	62	32	4	56	37	84	1	115	84	>200	1
3130xl	1	19	2	30	61	32	4	57	36	85	1	116	86	>200	2
3130xl	1	18	3	30	59	32	5	56	40	85	1	114	82	>200	2
3130xl	1	19	3	30	60	32	5	57	41	85	1	116	79	>200	2
3130xl	1	18	4	30	69	32	9	56	45	85	3	115	100	>200	3
3130xl	1	18	2	30	60	32	6	56	42	85	1	115	100	>200	2
3130xl	1	18	3	30	52	32	5	56	36	85	1	115	87	>200	2
3130xl	1	18	3	30	64	32	6	56	37	85	2	115	89	>200	2
3130xl	1	18	4	30	67	32	6	56	40	85	3	114	100	>200	3
3130xl	2	18	2	30	54	32	3	56	35	85	1	116	81	>200	1
3130xl	2	18	2	30	59	32	5	56	39	85	1	115	90	>200	2
3130xl	2	18	2	30	57	32	5	56	30	85	1	116	76	>200	2
3500xl	2	18	3	30	58	32	5	56	41	85	1	116	81	>200	1
3500xl	2	18	2	30	57	32	5	56	41	85	1	116	79	>200	2
3500xl	2	18	3	30	72	32	6	56	44	85	2	115	97	>200	2
3500xl	2	18	3	30	73	32	6	56	46	85	2	116	92	>200	3
Average			3%		62%		5%		39%		1%		88%		2%
Std. Dev.			1%		6%		1%		4%		1%		8%		1%

Supplemental Table S2. Cohort panel distribution of allele sizes and ARs determined using mPCR compared to the activation ratio from Southern blot analysis. Deidentified samples were processed at Asuragen using mPCR and compared to SB results. CGG repeat lengths are listed as the peak maxima associated with the normal allele and distribution of allele sizes for the premutation allele. ARs were determined on the normal allele for both mPCR and SB analysis.

Sample ID	CGG Repeats	mPCR Activation Ratio (%)	Southern blot Activation Ratio (%)
R01	30/86, 91	86	90
R02	30/98, 111, 120	90	90
R03	30/85, 89	3	0
R04	29/102, 116, 123	77	85
R05	20/74, 92, 95	72	75
R06	20/76, 95, 101	9	10
R07	31/79, 82	15	20
R08	29/92, 95	52	50
R09	35/106, 112	47	50
R10	31/63, 80, 83	50	50
R11	30/87, 104, 108	78	90
R12	20/93, 97	84	90
R13	20/93, 100	42	40
R14	23/118, 122-177, >200	48	60
R15	36/96, 98	33	20
R16	36/104, 108	0	10
R17	30/75, 77, 87, 90	57	50
R18	29/75, 78	71	75
R19	30/75, 88, 91	42	30
R20	20/93, 99	35	40
R21	27/91, 95	55	50
R22	35/96,99	35	20
R23	41/101, 105	58	60
R24	21/93, 95	46%	5
R25	29/92, 95	53	50

Supplemental Table S2 (*continued*).

Sample ID	CGG Repeats	mPCR Activation Ratio (%)	Southern blot Activation Ratio (%)
R26	20/75, 79	34	20
R27	23/118, 139, >200	39	50
R28	25/68, 80, 86	29	20
R29	25/90, 99	79	90
R30	27/66,68	16	20
R31	27/58,69,71	45	50
R32	30/72, 74	27	10
R33	30/79, 82	35	40
R34	20/91, 95	81	80
R35	30/102, 110	30	20
R36	29/77, 80	81	80
R37	32/76, 78	29	40
R38	30/94, 100	82	90
R39	30/94, 100	22	10
